# Intraoperative assessment and postsurgical treatment of prostate cancer tumors using tumor-targeted nanoprobes

**DOI:** 10.7150/ntno.50095

**Published:** 2021-01-01

**Authors:** James Teh, Manisha Tripathi, Derek Reichel, Bien Sagong, Ricardo Montoya, Yi Zhang, Shawn Wagner, Rola Saouaf, Leland W. K. Chung, J. Manuel Perez

**Affiliations:** 1 Department of Neurosurgery, Cedars-Sinai Medical Center, Los Angeles, CA 90048, USA.; 2 Biomedical Imaging Research Institute, Cedars-Sinai Medical Center, Los Angeles, CA 90048, USA.; 3 Department of Medicine, Uro-Oncology Research Program, Cedars-Sinai Medical Center, Los Angeles, CA 90048, USA.; 4 S. Mark Taper Foundation Imaging Center, Cedars-Sinai Medical Center, Los Angeles, CA 90048, USA.; 5 Samuel Oschin Comprehensive Cancer Institute, Cedars-Sinai Medical Center, Los Angeles, CA 90048, USA.; 6 Current address: Department of Cell Biology and Biochemistry, Texas Tech University Health Sciences Center, Lubbock, TX 79430, USA.

**Keywords:** image-guided therapy, near infrared fluorescence imaging, iron oxide nanoparticles, heptamethine cyanine, prostate cancer

## Abstract

Successful visualization of prostate cancer (PCa) tumor margins during surgery remains a major challenge. The visualization of these tumors during surgery via near infrared fluorescence (NIRF) imaging would greatly enhance surgical resection, minimizing tumor recurrence and improving outcome. Furthermore, chemotherapy is typically administered to patients after surgery to treat any missed tumor tissue around the surgical area, minimizing metastasis and increasing patient survival. For these reasons, a theranostics fluorescent nanoparticle could be developed to assist in the visualization of PCa tumor margins, while also delivering chemotherapeutic drug after surgery.

**Methods:** Ferumoxytol (FMX) conjugated to the fluorescent dye and PCa targeting agent, heptamethine carbocyanine (HMC), yielded the HMC-FMX nanoprobe that was tested *in vitro* with various PCa cell lines and *in vivo* with both subcutaneous and orthotopic PCa mouse models. Visualization of these tumors *via* NIRF imaging after administration of HMC-FMX was performed. In addition, delivery of chemotherapeutic drug and their effect on tumor growth was also assessed.

**Results:** HMC-FMX internalized into PCa cells, labeling these cells and PCa tumors in mice with near infrared fluorescence, facilitating tumor margin visualization. HMC-FMX was also able to deliver drugs to these tumors, reducing cell migration and slowing down tumor growth.

**Conclusion:** HMC-FMX specifically targeted PCa tumors in mice allowing for the visualization of tumor margins by NIRF imaging. Furthermore, delivery of anticancer drugs by HMC-FMX effectively reduced prostate tumor growth and reduced cell migration *in vitro*. Thus, HMC-FMX can potentially translate into the clinic as a nanotheranostics agent for the intraoperative visualization of PCa tumor margins, and post-operative treatment of tumors with HMC-FMX loaded with anticancer drugs.

## Introduction

Prostate cancer (PCa) remains one of the leading causes of death in men in the USA and around the world 1. Complete surgical resection of aggressive prostate tumors is typically performed to prevent further invasion into nearby organs or tumor metastasis to distal organs 2, 3. Surgeons typically use visual and tactical methods to differentiate between tumor and non-tumor tissue; however, these methods are not optimal as they could result in un-resected tumor tissue left after surgery. The problem is compounded by the close proximity of the prostate to other organs, limiting the use of wide surgical margins 4. Conventional imaging modalities such as X-ray computed tomography (CT), positron emission tomography (PET), magnetic resonance imaging (MRI) and ultrasound have been utilized to visualize cancerous tissues within the prostate before surgery for diagnosis and prognosis purposes 2, 5, 6. However, these imaging modalities are cumbersome to use in real time while in surgery to precisely identify tumor borders. Therefore, a fluorescence image-guided intraoperative approach may provide surgeons with a better method for objective, real-time visualization of PCa tumors during surgery 7. These intraoperative fluorescence methods operate within the near-infrared region (700-900 nm) penetrating deeper through blood and tissue with minimal tissue light scattering or light absorption by water and hemoglobin 8, 9. However, for this approach to be successful, molecules that absorb and emit fluorescence in the near infrared (NIR) region need to be specifically targeted to cancer tissue for proper identification of tumor margins during surgery.

Among the various types of NIR fluorescent dyes that can be used for cancer imaging, the heptamethine carbocyanine (HMC) dye not only exhibits strong NIR fluorescence, with excitation at 750 nm and emission at 800 nm, but also has high affinity to various cancer tissue, including those from liver, colon, brain, breast, lung and prostate 10-12. The specific targeting of HMC to cancer cells has been found to be mediated by the overexpression of organic anion transporting polypeptides (OATPs) in cancer cells. Given that the increased expression of OATPs is shared by multiple types of tumors, we hypothesized that the conjugation of HMC to the surface on FMX will facilitate drug delivery to tumors via OATP, while allowing for NIRF image-guided drug delivery. In PCa, the OATP1B3 and OATP1A2 subtypes have been shown to be overexpressed 10, 13-15. In particular, it has been demonstrated that the overexpression of OATP1B3 mediates the selective uptake of HMC ligands in PCa cells, but not in normal prostate epithelial cells. HMC has been shown to specifically target PCa tumors following intravenous administration, allowing for the fluorescence-based identification of these tumors in animal models 15. Furthermore, HMC-based drug conjugates have been developed as an image-guided drug delivery system to OATP-overexpressing PCa tumors 17, 18. However, these HMC-drug conjugates are limited in their drug delivery capabilities, as they demonstrate a maximum molar drug delivery capability of 1:1 (HMC:drug) and can be eliminated rapidly through renal clearance, minimizing the amount of drug reaching the tumor 17. In addition, synthesis of these conjugates involves further chemical modification of the drug for conjugation to the HMC dye, which could result in decreased potency of the drug. Therefore, an HMC-based nanoplatform technology could be developed for the fluorescence image-guided intraoperative resection of PCa tumor and the subsequent post-surgical image-guided delivery of drugs to remaining tumor tissue in the prostate and potentially metastatic lesions.

Herein we report the development of an HMC-based nanotheranostics platform for the intraoperative fluorescent visualization of PCa tumors as well as the postsurgical delivery of a drug to cancer tissues. At the core of our platform technology is Ferumoxytol (FMX), a 30 nm FDA-approved iron oxide nanoparticle clinically used for the treatment of anemia 19. FMX has increasingly been used off-label in MRI angiography due to its superparamagnetic properties 20-22. Human clinical trials have demonstrated the safety and ability of FMX as MRI contrast agent 23. Furthermore, studies in animal have shown that FMX can be used as a drug delivery nanovehicle allowing for MR-guided assessment of nanoparticle accumulation and drug release 24. To FMX, we conjugated HMC to develop an HMX-FMX nanoprobe for NIRF visualization of tumors during surgery and postsurgical delivery of chemotherapeutic drugs to un-resected PCa tumor tissue. We have recently reported the use of similar HMC-FMX nanoprobes for the visualization of glioblastoma multiform (GBM) and image-guided drug delivery across the brain-blood barrier in mice models 25. The resulting nanoprobe retained both the fluorescence properties of HMC and the magnetic properties of FMX, making it a magneto-fluorescent nanoprobe that targeted GBM tumors in intracranial mouse models. Herein, we expand these studies toward the detection of PCa tumor margins and drug delivery. We evaluated the intraoperative capabilities of these nanoprobes using a Synchronized near-InfraRed Imaging System (SIRIS), which was developed in-house to detect NIR imaging agents *in vivo* 26. By comparing the NIRF nanoprobe-based imaging of PCa tumors with traditional immunohistochemical analysis, the accuracy of HMC-FMX for intraoperative PCa tumor detection was determined. In addition, the therapeutic capabilities of HMC-FMX were assessed by loading docetaxel (DXT) into FMX and testing its effect to cancer cell viability and cell migration *in vitro* as well as tumor growth *in vivo*. Finally, we further investigated the therapeutic capabilities of HMC-FMX using cabozantinib (CZT), a tyrosine kinase inhibitor that has shown promise in treating PCa.

## Methods

### Materials

Ferumoxytol (FMX) was obtained from AMAG Pharmaceuticals (Waltham, MA). Docetaxel (DXT) and Cabozantinib (CZT) were purchased from LC Laboratories (Woburn, MA). HMC dye was synthesized as previously described 27, 28. Dialysis tubing (6 ~ 8 kDa MWCO) was purchased from Spectrum Laboratories (Rancho Dominguez, CA). Deionized water (DI H_2_O) was obtained using a Milli-Q reverse osmosis system (MilliporeSigma, Burlington, MA). N-hydroxysuccinimide (NHS), poly-D-lysine hydrobromide, sodium azide (NaN_3_) and centrifugal filter unit (Amicon® Ultra-4, 3 kDa MWCO) were purchased from Sigma Aldrich (St Louis, MO). 1-ethyl-3-[3-dimethylaminopropyl]carbodiimide hydrochloride (EDC), dimethyl sulfoxide (DMSO), acetonitrile (MeCN), paraformaldehyde (PFA), oligomycin, 2-deoxy-D-glucose (2-DG), rifampicin, cyclosporin A (CsA), cholecystokinin octapeptide (CCK-8), crystal violet, sodium dodecyl sulfate (SDS), Annexin V binding buffer (10X), and formalin solution (10%) were purchased from Fisher Scientific (Pittsburgh, PA). 4',6-diamidino-2-phenylindole (DAPI), TrypLE™ Express (1X), Slide-A-Lyzer™ MINI dialysis cups (3.5 kDa MWCO), and BupH™ MES buffered saline packs were obtained from ThermoFisher Scientific (Waltham, MA). 7-amino-actinomycin D (7-AAD), phycoerythrin annexin V (PE annexin V) and Matrigel were purchased from BD Biosciences (San Jose, CA). 8.0 μm pore size Transwell™ permeable inserts were obtained from Corning Inc (Corning, NY). All chemicals and reagents were purchased and used without further purification.

### Cell Lines

PCa cells LNCaP, 22Rv1, PC3 and DU145 and healthy prostate cells RWPE-1 were obtained from American Type Culture Collection (ATCC, Manassas, VA). Cell media RPMI-1640 and Keratinocyte-SFM (K-SFM) were acquired from Fisher Scientific (Pittsburgh, PA). Fetal Bovine Serum (FBS) was purchased from Atlanta Biologicals (Flowery Branch, GA). Antibiotics-Antimycotic solution (AA) was purchased from Thermo Fisher Scientific (Waltham, MA). LNCaP, 22Rv1, PC3 and DU145 cells were cultured in RPMI-1640 media supplemented with 10% FBS and 1% AA. RWPE-1 cells were cultured in K-SFM media supplemented with bovine pituitary extract (50 μg/mL), human epidermal growth factor (5 ng/mL) and 1% AA. All cell lines were maintained in a humidified atmosphere at 37 °C with 5% CO_2_ air.

### Preparation of HMC-FMX Nanoprobes

**Scheme 1:**
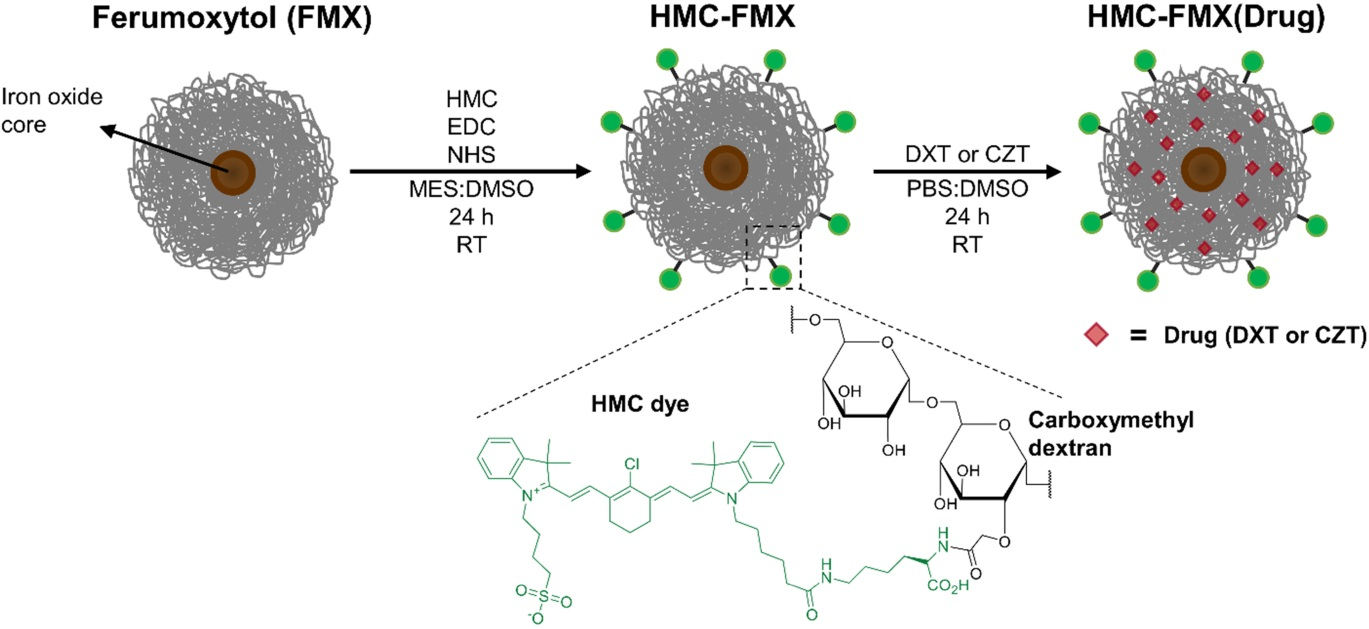
Preparation of Ferumoxytol (FMX) functionalized with near-infrared heptamethine carbocyanine (HMC) dyes and loaded with anticancer drugs.

HMC was conjugated to the carboxymethyl dextran-coated FMX by forming an amide bond between carboxyl groups on dextran and amine groups of HMC dye using an EDC and NHS coupling procedure (**Scheme 1**). Briefly, 100 μL of FMX (30 mg/mL of Fe) to 200 μL of 0.1 M MES buffer (pH 6). Then, EDC (13.0 mg, 67.8 μmol) and NHS (2.1 mg, 18.2 μmol) were added to this solution. HMC dye (1.0 mg, 1.2 μmol) was dissolved in 250 μL of DMSO and added dropwise to the reaction solution. The reaction solution was stirred for 24 h at RT in the dark, and subsequently purified by dialysis with frequent changes of DI H_2_O, then a final dialysis with PBS for 4 h. The purified HMC-FMX was collected, diluted to 1.67 mg/mL [Fe] or 30 mM [Fe] concentration with PBS and stored at 4 °C in the dark for up to three months before use. The number of HMC dyes conjugated to FMX was quantified. Briefly, a solution of HMC-FMX was dissolved in a 1:1 PBS:MeCN mixture and iron was precipitated and analyzed using a SpectraMax M5 plate reader (Molecular Devices, San Jose, CA). The dye absorbances (785 nm) of HMC-FMX were compared to the absorbance of standard solutions in a HMC calibration curve. In addition, the absorbance spectra of HMC and HMC-FMX (equimolar dye) were compared.

### Drug Loading into HMC-FMX Nanoprobes

The encapsulation of drugs into FMX or HMC-FMX nanoprobes were conducted by a solvent-diffusion method 29. Briefly, stock solutions of DXT or CZT were prepared in DMSO (20 mg/mL). Then, a DXT or CZT solution (200 uL) was added dropwise over a period of 1 minute to either FMX or HMC-FMX nanoprobe solutions (1.67 mg/mL [Fe]) under vortex (1,500 *rpm*) at RT. The drug-loaded nanoprobes were mixed for 24 h at RT to facilitate the entrapment of hydrophobic molecules within the FMX coating. Then, the drug-loaded FMX or HMC-FMX nanoprobes were centrifugated using an Amicon Ultra-4 centrifugal filter unit at 3,300 *rpm* for 40 min to remove free drug and DMSO. The drug-loaded FMX or HMC-FMX nanoprobes were collected, diluted to 1.67 mg/mL [Fe] concentration with PBS and stored at 4 °C in the dark.

### Drug Loading and Encapsulation Efficiency Measurement

The drug loading and encapsulation efficiency of HMC-FMX nanoprobes was analyzed by high performance liquid chromatography (HPLC). Briefly, 100 uL solutions of DXT- or CZT-loaded HMC-FMX nanoprobes were added to 300 μL mixture of MeCN and PBS at pH 4.0 (2:1, v/v), and incubated for 24hours at RT to precipitate iron and release the entrapped drugs. Then, the samples were centrifuged at 3,300 *rpm* for 5 min, and the supernatants were collected for HPLC analysis (Agilent Technologies 1260 Infinity, Santa Clara, CA) equipped with an Apollo™ C18 column (Hichrom, Leicestershire, UK) using the following conditions: flow rate, 1.0 mL/min; injection volume, 20 μL; detection wavelength, 230 nm. Analysis of the released drugs was determined with an isocratic method with a mobile phase consisting of 75% (MeCN) and 25% (Water). Drug concentrations in nanoprobes were determined by comparing HPLC peaks with a standard calibration curve prepared from known concentration standards.

### Drug Release from HMC-FMX Nanoprobes

The release of DXT from HMC-FMX nanoprobes was conducted by a dialysis method. Briefly, 1.5 mL of HMC-FMX(DXT) solution (1.67 mg/mL [Fe]) was added to a presoaked dialysis cup. The cup was placed into 15 mL of dialysis solution containing PBS at pH 6.8, or 7.4 with 20% FBS. The nanoprobe solution was dialyzed for 48 h at 37 °C. Aliquots (200 µL) were removed at 0, 1, 3, 6, 24 and 48 h, and concentrations of DXT were measured by HPLC as described previously. Dialysis solution was replaced each time the nanoprobe solution was removed at specific time points.

### Characterizations of Nanoprobes

The size and surface charge of nanoprobes were analyzed by dynamic light scattering (DLS) using a Zetasizer Nano ZS90 (Malvern Instruments, Malvern, UK). FMX was diluted to 1.67 mg/mL [Fe] in PBS, while HMC-FMX and HMC-FMX(DXT) were diluted to 0.056 mg/mL [Fe] in PBS to prevent sample absorbance interference with the instrument laser. The magnetic resonance (MR) relaxation potential of HMC-FMX and HMC-FMX(DXT) were investigated using a 9.4 T Preclinical Bruker Biospin (Billerica, MA). Briefly, various concentrations of FMX, HMC-FMX and HMC-FMX(DXT) were prepared by diluting in DI H_2_O. After measuring the relaxation times of each FMX, HMC-FMX and HMC-FMX(DXT) samples, the longitudinal relaxation rate r_1_ (1/T_1_, s^-1^) and transversal relaxation rate r_2_ (1/T_2_, s^-1^) were calculated. In addition, the T_2_ detection of nanoprobes-treated cells were determined by treating LNCaP cells with 1.67 mg/mL [Fe] of each nanoprobe and collecting nanoprobe-treated cell pellets of: 100×10^3^, 50×10^3^, 10×10^3^ and 5×10^3^ cells. Absorbance and fluorescence of HMC-FMX and HMC dye (2.5 μg/mL [HMC] with PBS) was also measured using a UV/Vis spectrophotometer (Evolution 201, ThermoFisher Scientific, Waltham, MA) and a fluorescence spectrophotometer (LS 55, PerkinElmer, Waltham, MA), respectively.

### Fluorescence Detection Limit of HMC-FMX Nanoprobes in Cultured Cells

The fluorescence detection limits of HMC-FMX were examined in two separate experiments. First, to determine a concentration-based detection limit of HMC-FMX in large cell pellets, LNCaP cells were seeded and incubated overnight at 37 °C. The cells were treated with HMC-FMX at 0.03 μg/mL, 0.3 μg/mL, 3.0 μg/mL and 30.0 μg/mL [HMC]. After 24 h, media was removed, and the cells were harvested by trypsinization. Cells were centrifuged at 1300 *rpm* for 3 min and rinsed twice with PBS. Fresh media was gently added to the pelleted cells that were collected in 1.5 mL microtubes and imaged by SIRIS. Second, to determine a cell-based detection limit at the concentration-based limit found previously, we seeded LNCaP cells at a density of 100, 50, 10, 5, 1, 0.5, and 0.2×10^3^ cells/well and incubated cells with 0.3 μg/mL HMC-FMX. After 24 h, media was removed, and the cells were harvested by trypsinization. Cells were centrifuged at 1300 *rpm* for 3 min and rinsed twice with PBS. Fresh media was gently added to the pelleted cells that were collected in 1.5 mL microtubes and imaged by SIRIS.

### *In vitro* Assessment of HMC-FMX Nanoprobes Uptake

The cellular uptake of HMC-FMX was evaluated using NIR fluorescence imaging and flow cytometry. For NIR fluorescence imaging, 22Rv1, PC3, DU145 and RWPE-1 cells were seeded in a 24-well plate, while LNCaP cells were seeded in a poly-D-lysine coated 24-well plate at a density of 70×10^3^ cells/well. For flow cytometry studies, 22Rv1 and PC3 cells were cultured in a 6-well plate at a density of 250×10^3^ cells/well.

#### *In vitro* cellular uptake of HMC-FMX

After culturing 22Rv1, PC3, DU145, LNCaP and RWPE-1 cells overnight at 37 °C, the cells were treated with HMC-FMX at 8.0 μg/mL [HMC] for 3 h. Medium was removed from the wells and rinsed twice with PBS. Cells were then fixed with 4% PFA at RT for 10 min and stained with DAPI (300 nM in PBS) for 15 min at RT. The cells were imaged using a fluorescence microscope (Keyence BZ-X710, Keyence, Osaka, Japan).

#### Time-dependent uptake of HMC-FMX

After culturing 22Rv1 cells overnight at 37 °C, the cells were treated with HMC-FMX at 8.0 μg/mL [HMC] and incubated at various time points: 0.5, 3, or 6 hours. Media was removed from the wells and rinsed twice with PBS. Cells were then fixed with 4% PFA at RT for 10 min and stained with DAPI for 15 min at RT. The cells were imaged using a fluorescence microscope.

For flow cytometric study, 22Rv1 cells were cultured overnight at 37 °C, followed by treatment with HMC-FMX at 8.0 μg/mL [HMC]. After incubating at various time points: 0.5, 3, or 6 hours, the medium was removed, and the cells were harvested by trypsinization. Then, cells were rinsed twice with PBS and fixed with ice-cold 4% PFA for 15 min. Cells were resuspended in FACS buffer (1% FBS and 0.05% NaN_3_ in PBS) and analyzed by LSR Fortessa flow cytometry (BD Biosciences, San Jose, CA) to record the fluorescence histograms of HMC dyes.

#### Uptake mechanism studies of HMC-FMX

OATP-mediated uptake of HMC-FMX was examined. 22Rv1 cells were pretreated with OATP inhibitors rifampicin (25 μM), CsA (20 μM) and CCK-8 (20 μM) for 3 h at 37 °C. Then, the medium was removed, and cells were treated with HMC-FMX at 8.0 μg/mL [HMC] for 3 hours at 37 °C. Medium was removed from the wells, and the cells were washed once with PBS. Next, cells were fixed with 4% PFA at RT for 10 min and stained with DAPI for 15 min at RT. The cells were imaged using a fluorescence microscope. In addition, uptake mechanism studies of HMC-FMX treated with 22Rv1 cells were analyzed by flow cytometry. Briefly, after pretreating 22Rv1 cells with different OATP inhibitors, HMC-FMX at 8.0 μg/mL [HMC] was added to the cells and incubated for 3 hours. Media was removed, and the cells were harvested by trypsinization. Cells were resuspended in FACS buffer and analyzed by LSR Fortessa flow cytometry to record the fluorescence histograms of HMC dyes.

### *In vitro* Cytotoxicity Assay

The cell viability of multiple PCa cell lines was analyzed using a cell adhesion assay. 22Rv1, PC3 and DU145 cells (10×10^3^ cells/well) were plated in a 96-well plate, while LNCaP cells (10×10^3^ cells/well) were plated in a poly-D-lysine coated 96-well plate. After 24 h, cells were treated with various concentrations of HMC-FMX(DXT), FMX(DXT) or DXT. After 72 h, media was removed, and the wells were rinsed once with PBS. The cells were fixed with 4% PFA at RT for 10 min, followed by rinsing with PBS. Cells were treated with 100 μL of crystal violet solution (0.5 mg/mL in DI H_2_O) and incubated at RT for 20 min. Then, the crystal violet solution was removed, and the wells were rinsed twice with DI H_2_O (100 μL/well). SDS solution (2% in PBS) was added (100 μL/well) and incubated for an additional 30 min at RT to dissolve the crystals. The absorbance of the wells was acquired at 595 nm using a microplate reader (SpectraMax Plus 384, Molecular Devices).

### *In vitro* Cell Migration Assay

The migration assay of LNCaP and PC3 cells was performed as previously described 30, 31. Briefly, LNCaP or PC3 cells (20×10^3^ cells/well) were added to the upper wells and incubated for 4 h at 37 ºC to allow cells to adhere to the membrane. Media in the upper wells were replaced with serum-free media mixed with either 10 nM DXT or HMC-FMX(DXT) with an equivalent amount of DXT, while the bottom wells were replenished with fresh media containing serum. After incubating for 24 h at 37 ºC, any cells that were remaining on the upper filters were scraped off gently with a cotton swab, and the inserts were washed with PBS. The migrated cells (bottom chamber) were fixed with 4% PFA for 10 min and stained with crystal violet solution (0.5 mg/mL in DI H_2_O) for 20 min at RT. The total numbers of migrated cells were counted manually based on five images, which were randomly captured for each insert with a fluorescence microscope (Keyence BZ-X710, Keyence, Osaka, Japan).

### *In vitro* Cell Apoptosis Assay

22Rv1 cells were seeded in a 6-well plate at a density of 250×10^3^ cells/well and incubated overnight at 37 °C. The cells were treated with HMC-FMX(DXT), HMC-FMX, or PBS. After 72 h, media was removed from the wells, and the cells were harvested by trypsinization. Cells were rinsed twice with PBS and resuspended in 400 μL of binding buffer (1X). The cell suspensions were treated with 5 μL of 7-AAD and PE annexin V, followed by incubation in the dark at RT for 15 min. The percent of early apoptosis and that of late apoptosis were measured by LSR Fortessa flow cytometry (BD Biosciences, San Jose, CA).

### Animal Models

Mouse experiments were conducted in strict compliance with the protocols approved by the Institutional Animal Care and Use Committee (IACUC), Cedars-Sinai Medical Center. Three- to four-week-old nu/nu male mice were obtained from Charles River (Hopkinton, MA). Animals were housed in a pathogen free environment and were given sterilized water and rodent chow. For implantation of subcutaneous prostate tumors in mice, PC3 or 22Rv1 cells were suspended in a 1:1 mixture of cell media and Matrigel (10×10^6^ cells/mL). Then, the mice were injected subcutaneously with either PC3 or 22Rv1 cells (1×10^6^ cells/100 μL) on the left and right flank. Experiments with tumor-bearing mice were performed when the tumors had reached a volume of about 100 mm^3^. For orthotopic implantation of prostate tumors in mice, cells were grafted in the anterior lobes of the prostate of 8 week old mice, as previously described 32. Experiments were performed on tumor-bearing mice a week after surgery.

### *In vivo* Targeted Imaging of PCa in Mice

In the case of the subcutaneous prostate tumor-bearing mice, a solution of HMC-FMX in PBS (7 mg [Fe]/kg) was injected intravenously. After 72 h post-injection, mice were euthanized. The brain, heart, lung, liver, kidneys, spleen and tumors were excised from the mice. Fluorescence images of mice, organs and tumors were obtained using an *In vivo* Imaging System (IVIS, PerkinElmer, Waltham, MA). In the case of the orthotopic prostate tumor-bearing mice, a solution of HMC-FMX in PBS (7 mg [Fe]/kg) was injected intravenously. After 72 h post-injection, mice were anesthetized. The whole-body imaging was performed using an IVIS or a SIRIS to evaluate the ability of HMC-FMX to fluorescently labeled prostate tumor. For intraoperative and post-operative visualization, the SIRIS camera was used to locate the fluorescently labeled prostate tumor. After the mouse was euthanized, an incision was made on the abdominal cavity exposing the prostate gland along with the fluorescently labeled tumor. The prostate gland-containing tumor was carefully excised from the mouse. The MR capability of HMC-FMX was examined *in vivo*, after 72 h post-injection. T_2_-weighted images were collected using a 9.4T horizontal bore magnet (Bruker Biospin MRI, Billerica, MA).

### *In vivo* Therapeutic Efficacy Studies

After the subcutaneous prostate tumor reached an average volume of 100 mm^3^, the mice were intravenously injected with 100 μL of either PBS, *Drugs*, or HMC-FMX (*Drugs*), where the *Drugs* represent DXT or CZT. Each treatment group contained 6 animals. The injected dose was 0.5 mg [*Drugs*]/kg. Tumor volume was measured with a caliper every 3 days during the period of treatment (31 days), where the volume was calculated by the formula: width (*w*) × height^2^ (*h^2^*) ×* 1/2*.

### Histological Examinations

At the end of the experiment, the tumors and organs from each group of mice were harvested and fixed in formalin solution, followed by 70% EtOH. The tumors and organs were sent to the Biobank and Translational Research Core at Cedars-Sinai Medical Center for tissue sectioning and hematoxylin and eosin (H&E) staining. Brightfield images of H&E-stained tissue sections were visualized under a fluorescence microscope for any evident sign of toxicity and accumulation of HMC-FMX by fluorescence distribution on the tissue.

### Statistical Analysis

Measurements were performed in triplicate and reported as mean ± standard deviation, unless otherwise noted. We applied One-way ANOVA to compare significant differences between measurements. *P* values as **p < 0.01, ***p < 0.001, and ****p < 0.0001 were indicated in figures. Statistics and fitting of experimental data were performed with Prism 7 (GraphPad, San Diego, CA).

## Results

### Preparation and characterization of HMC-FMX

We prepared the HMC-FMX nanoprobe by conjugating the lysine portion of HMC to carboxylic acid groups on FMX's carboxymethyldextran coating using an EDC/NHS coupling method (**Scheme 1**). Dynamic light scattering (DLS) measurements show that the as-synthesized HMC-FMX has an average diameter of 37.0 ± 3.0 nm, PDI of 0.10 ± 0.01 and zeta potential of -11.8 ± 0.3 mV (**Table 1**). The resulting HMC-FMX was found to have an average of 40 HMC molecules per FMX nanoparticle.

**Figure 1:**
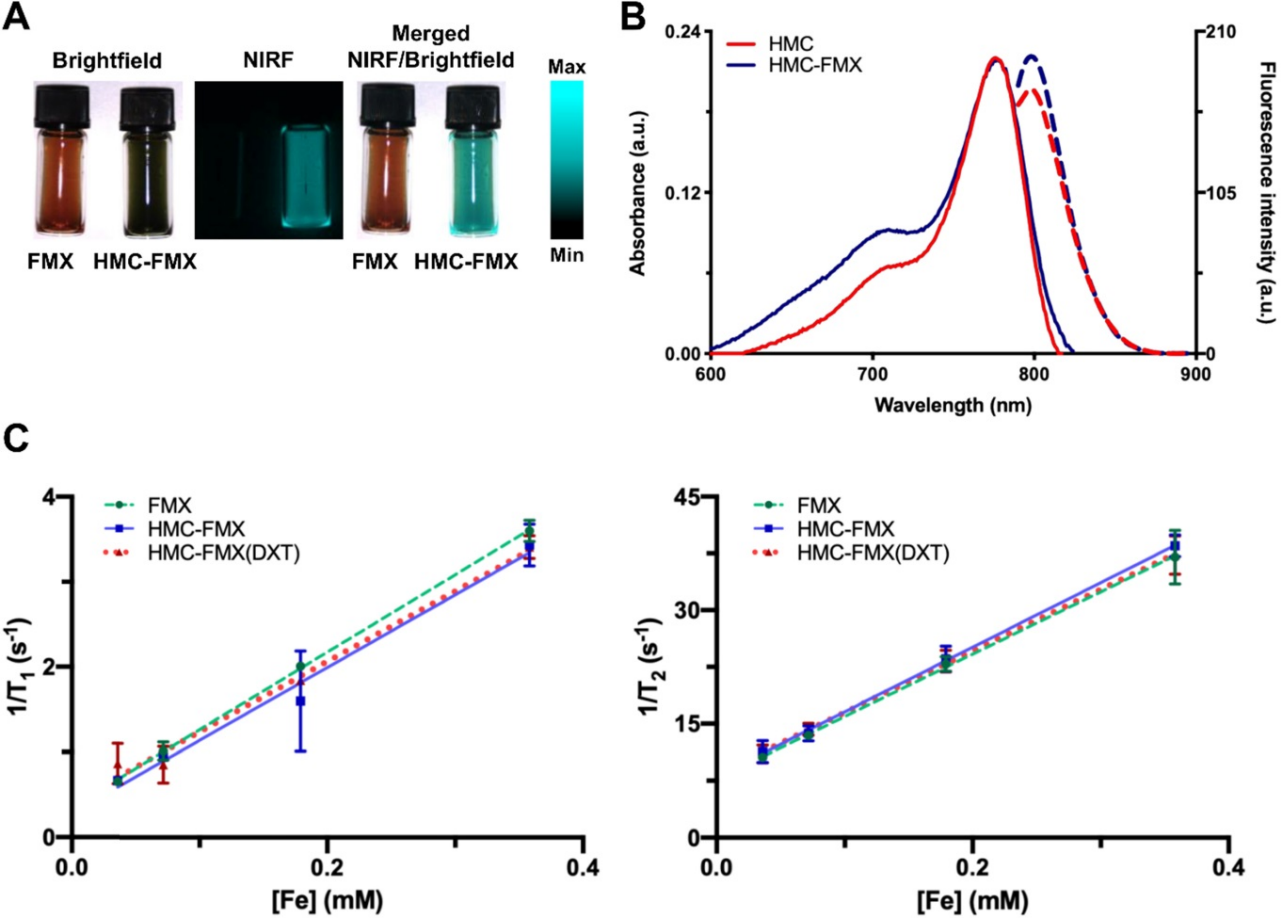
** Near infrared fluorescence (NIRF) and magnetic resonance (MR) characterizations of HMC-FMX.** Representative brightfield, NIRF, and merged (NIRF/brightfield) images of HMC-FMX and FMX in PBS (**A**). Absorbance (solid lines) and fluorescence (dashed lines) spectra of HMC-FMX and HMX dye (**B**). r_1_ and r_2_ relaxivity measurements of nanoprobes (**C**).

The NIRF emission of HMC-FMX is an important property for *in vivo* PCa intraoperative detection. To examine HMC-FMX fluorescence, we used the Synchronized near-Infrared Imaging System (SIRIS), a preclinical NIRF imaging system 24, 31. The SIRIS operates as a device that fluorescently excites clinically approved NIR fluorophore indocyanine green (ICG), and a camera system that records and captures high-definition images and videos, with and without excited NIRF. As a preclinical NIRF imaging system, SIRIS enabled intraoperative detection and resection of tumors labeled with NIR fluorophores 24, 31. Brightfield imaging revealed a color change from brown to dark green, indicating successful labeling of HMC to FMX (**Figure 1A**). In addition, NIRF imaging showed bright and stable fluorescence emission by HMC-FMX under NIR excitation at 785 nm by SIRIS (**Figure 1A**). Furthermore, we evaluated absorbance and fluorescence properties of HMC after conjugating to FMX, in comparison to HMC alone at equimolar concentrations. The ultraviolet/visible (UV/Vis) absorption spectra showed both HMC-FMX and HMC exhibited a peak at 780 nm (**Figure 1B**). Moreover, HMC-FMX exhibited NIRF emission at 800 nm, with a moderate increase in fluorescence intensity over HMC (**Figure 1B**). These results show that HMC conjugation to FMX does not affect the size or polydispersity of the FMX nanoparticle preparation, or the NIRF properties of HMC.

We then investigated the magnetic resonance (MR) signal of FMX, after HMC conjugation by measuring T_1_ and T_2_ relaxation times under different Fe concentrations. Results show that the relaxation rate (1/T_1_ and 1/T_2_) were linearly correlated with HMC-FMX concentrations (**Figure 1C**). Conjugation of HMC and subsequent encapsulation of DXT into FMX does not dramatically change either the r_1_ or r_2_ relaxivities, or r_2_/r_1_ ratios of FMX. (**Table S1**). r_2_ relaxivities were ~80-85 mM-1s-1, while r_2_/r_1_ ratios were ~9-10 for all FMX nanoprobes. These findings indicate that the magnetic relaxation properties of FMX are unaffected after conjugating HMC to FMX.

### HMC-FMX internalizes and localizes in prostate cancer cells

**Figure 2:**
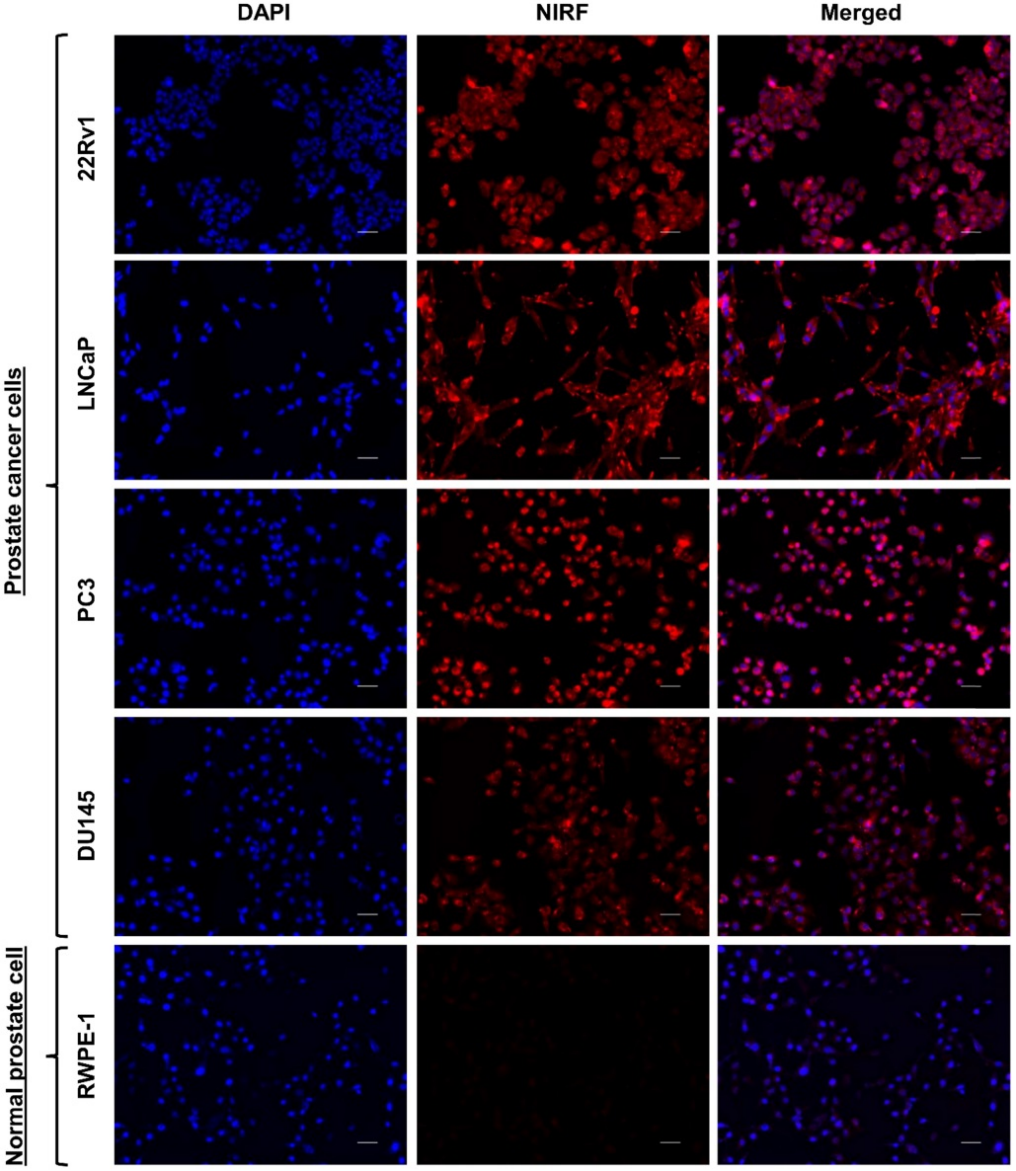
** Cellular uptake of HMC-FMX in PCa cells, compared to normal prostate epithelial cells.** Fluorescence microscope images of PCa cells: 22Rv1, LNCaP, PC3, and DU145, and normal prostate cell: RWPE-1, treated with HMC-FMX for 3 h. HMC-FMX selectively accumulates and localizes in PCa cells. For each panel, the fluorescence images from left to right show nuclei stained by DAPI (blue), cells stained by HMC-FMX (red) and overlay of the two images. Scale bars are 50 µm. Magnification: 20×.

We first evaluated whether the generated HMC-FMX could discriminate between PCa cells and normal cells. NIRF imaging showed accumulation of HMC-FMX fluorescence in multiple PCa cell lines: 22Rv1, LNCaP, PC3, and DU145 (**Figure 2**). In contrast, NIRF imaging of normal prostate epithelial RWPE-1 cells revealed negligible accumulation of HMC-FMX fluorescence (**Figure 2**). As HMC-FMX could discriminate between prostate cancer and normal cells, we examined HMC-FMX cellular uptake efficacy in PCa cells. 22Rv1 cells were treated with HMC-FMX for various time intervals. NIRF imaging showed intracellular fluorescence accumulation in 22Rv1 cells increased gradually with incubation time from 30 min to 6 h (**Figure S1A**). Flow cytometric analysis revealed distribution of HMC-FMX fluorescence, with increased uptake efficiency, and significant fluorescence intensity measured at 6 h (**Figures S1B and S1C**). These findings indicate that HMC-FMX can potentially target PCa cells, leading to intracellular accumulation of HMC-FMX fluorescence over time.

Encouraged by these results, we further analyzed the HMC-FMX fluorescence emission and T_2_ relaxation upon internalization in PCa cells. First, we examined the lowest concentration of HMC-FMX inside LNCaP cells that can be detected by NIRF, by treating cells with different concentrations of HMC-FMX. NIRF imaging by SIRIS revealed that the lowest HMC-FMX fluorescence emission was at 0.3 μg/mL [HMC] inside an LNCaP cells pellet (10×10^3^ cells/vial) (**Figure S2A**). Subsequently, we examined the HMC-FMX fluorescence emission by treating different LNCaP cell densities with HMC-FMX at 0.3 μg/mL [HMC]. NIRF imaging by SIRIS exhibited HMC-FMX fluorescence emission at 5×10^3^ cells (**Figure S2B**). The MR signal of HMC-FMX was investigated with different LNCaP cell densities treated at HMC-FMX at 0.3 μg/mL [HMC]. The T_2_ relaxation time linearly correlated with LNCaP cell densities, which affected MR contrast change at higher LNCaP cell densities (**Figure S2C**). These data indicate that one can still detect HMC-FMX by fluorescence or magnetic relaxation even upon internalization into PCa cells.

We next hypothesized that the observed HMC-FMX uptake is driven by OATP. Thus, we preincubated 22Rv1 cells with known OATP inhibitors (CsA, rifampicin, or CCK-8) before incubation with HMC-FMX and observed the level of cell-associated fluorescence by microscopy 24 hours after incubation. Results showed that preincubation of 22Rv1 cells with OATP inhibitors reduced the degree of HMC-associated fluorescence in these cells by fluorescence microscopy (**Figure S3A**). Furthermore, the calculated average fluorescence intensity of cells pre-incubated with either rifampicin or CCK-8 resulted in a three-fold decreased in NIRF intensity (**Figure S3B**). Taken together, these results suggest that the uptake of HMC-FMX is driven by OATP, similarly to what has been reported for HMC.

### HMC-FMX localizes specifically to PCa tumors in xenograft and orthotopic mouse models

**Figure 3:**
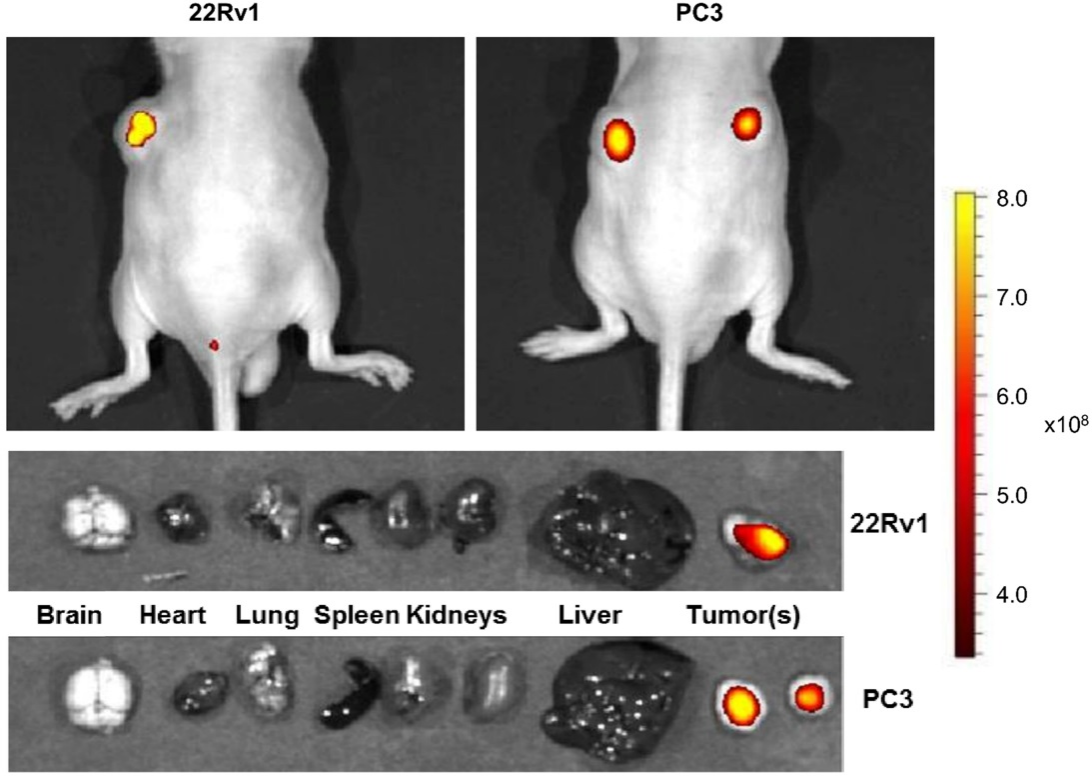
**HMC-FMX targets PCa tumors in xenograft mouse models.** Representative NIRF images of mice bearing subcutaneous 22Rv1 and PC3 tumors 72 h after injection with HMC-FMX shows sensitive NIRF detection of tumors to facilitate intraoperative resection of PCa tumors. NIRF images of excised organs and HMC-FMX fluorescently labeled tumors - 22Rv1 and PC3 - acquired at 72 h post-injection.

To test the effectiveness of HMC-FMX for *in vivo* imaging, we intravenously administered HMC-FMX via tail vein injection into nude mice implanted with subcutaneous 22Rv1 or PC3 PCa prostate cancer tumors. NIRF imaging by IVIS revealed brightly fluorescence labeled 22Rv1 or PC3 PCa tumors by HMC-FMX, after 72 h post-injection (top panel,** Figure 3**). The excised organs and tumors were imaged using the same thresholding value as the whole body images. HMC-FMX fluorescence emission signal was observed only within the tumors at this thresholding value (bottom panel, **Figure 3**). When organs were imaged using different thresholding values to increase sensitivity, fluorescence signal was observed in the rest of the organs, while the fluorescence signal was saturated in the tumors (**Figure S4**). These results indicate that the fluorescence signal of HMC-FMX is higher in the tumors as opposed to the rest of the other organs, suggesting the use of HMC-FMX for visualization of tumors during surgery.

**Figure 4:**
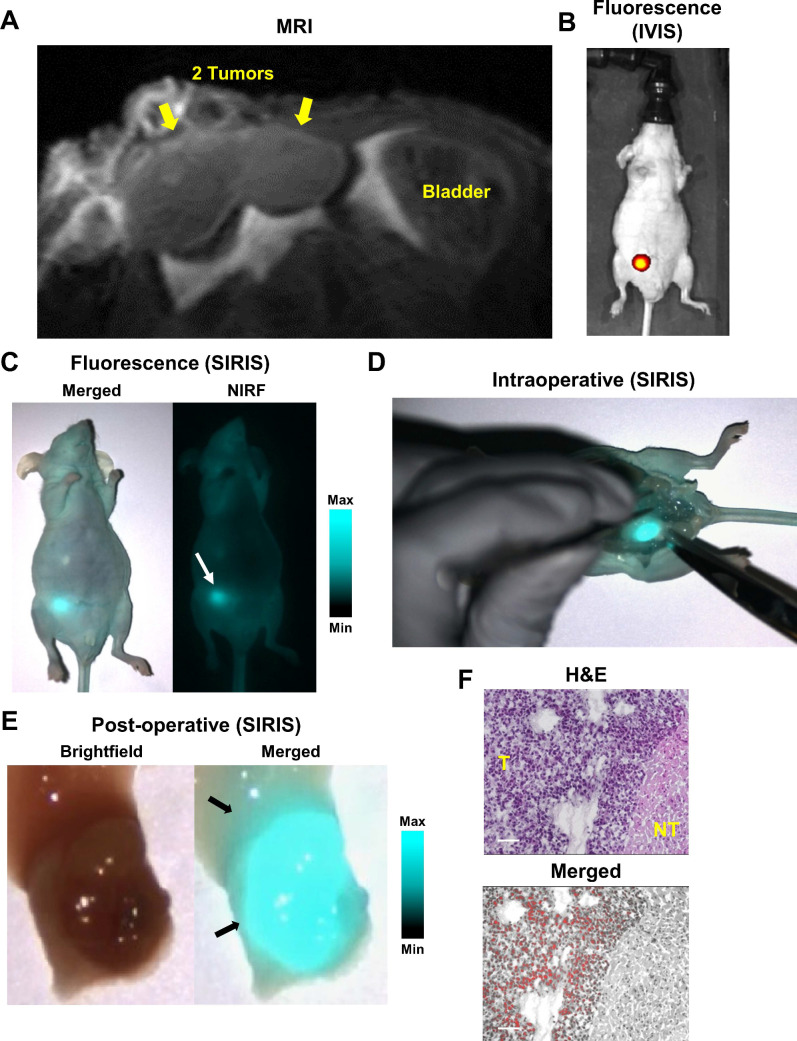
** HMC-FMX targets orthotopic prostate tumor in mice.** MR image of whole mouse shows tumors on the right anterior lobe of the mouse prostate 72 h after injection of HMC-FMX (**A**). Yellow arrows represent tumors. NIRF image of whole mouse 24 h after injection of HMC-FMX shows NIRF detection of primary tumor in the mouse prostate's right anterior lobe (**B**). Representative merged (NIRF/brightfield) and NIRF images of whole mouse show localization of HMC-FMX (**white arrow**) using an NIR imaging system, SIRIS (**C**). Intraoperative detection shows the fluorescently labeled primary tumor (**D**). Merged (NIRF/brightfield) image of the resected mouse prostate shows delineation of the primary tumor and healthy tissue, as indicated by black arrows (**E**). H&E staining and merged (NIRF/brightfield) images of the tissue section at the interface of normal tissue (NT) and tumor (T). HMC-FMX fluorescence (red) is detected inside the tumor region (**F**). Scale bars are 100 µm.

We further evaluated HMC-FMX for intraoperative detection *in vivo* using an orthotopic 22Rv1 PCa nude mouse model. After administering HMC-FMX intravenously, we recorded T_2_-weighted images, 72 hours post injection, to assess the location and shape of the PCa tumor. MR images of the mouse right anterior prostate lobe indicated the presence of a bilobal tumor (**Figure 4A**). NIRF imaging using IVIS and SIRIS imaging systems, showed localized HMC-FMX fluorescence at the prostate lobe (**Figures 4B and 4C**). After confirming the location of the primary tumor, we performed a mock surgery to remove the prostate. Under SIRIS, the lower abdominal area of the mouse was exposed, revealing a bright fluorescence signal of the prostate lobe (**Figure 4D**). Fluorescence was clearly associated with the implanted tumor and not with the nearby tissue, indicating specific tumor targeting. The resected NIR-labeled tumor was imaged using the SIRIS camera (**Figure 4E**), revealing a fluorescent tumor with a bilobal structure as seen in MRI (**Figure 4A**). Histopathological analysis of a resected tumor area, where differentiated tumor (T) and non-tumor (NT) margins on H&E staining are clearly identified, showing specific localization of HMC fluorescence only in the tumor (T) area, while no fluorescence is observed in the non-tumor (NT) area (**Figure 4F**). Taken together, these results show that HMC-FMX specifically associates with PCa tumors in both a subcutaneous and orthotopically implanted PCa mouse model, fluorescently labeling its tumor margins, with minimal association with non-tumor tissue.

### HMC-FMX delivers anticancer drugs to PCa cells

To test whether HMC-FMX can perform as an anticancer drug delivery vehicle, we loaded docetaxel (DXT) to HMC-FMX. The use of DXT in PCa has delayed cancer progression, particularly in castration-resistant PCa patients (CRPC) 33-35. Unfortunately, DXT has a low aqueous solubility, which requires the use of polysorbate-80 formulations to deliver to tumors. In addition, DXT formulations demonstrate poor tumor accumulation when administered systemically. Dose escalation in animal and humans is challenging due to its neurotoxicity when administered systemically. For these reasons, nanoparticle formulations have been proposed to deliver DXT and other drugs in high amounts to tumors 36-41.

Encapsulation of DXT did not dramatically change the magnetic relaxation (r_1_ or r_2_), diameter, zeta potential or polydispersity of the HMC-FMX nanoparticles. (**Table S1-S2**). The average percent encapsulation and loading of DXT in FMC-FMX were found to be 62% and 30%, respectively (**Table S2**). We next analyzed the rate of DXT release in acidic (pH 6.8) and neutral (pH 7.4) PBS with 20% serum. At different time intervals, the release of DXT at pH 6.8 and pH 7.4 in 20% serum was quantified by high-performance liquid chromatography (HPLC). The rate of DXT release at pH 7.4 with 20% serum was gradual with a t_1/2_ of 48.7 h, in comparison to the rate of DXT release at pH 6.8 with a t_1/2_ of 13.4 h (**Figure S5**). As PCa tumors are slightly acidic, the faster rate of drug release at pH 6.8 suggest that upon reaching the tumor HMC-FMX(DXT) would release the drug faster, than while in circulation.

**Figure 5:**
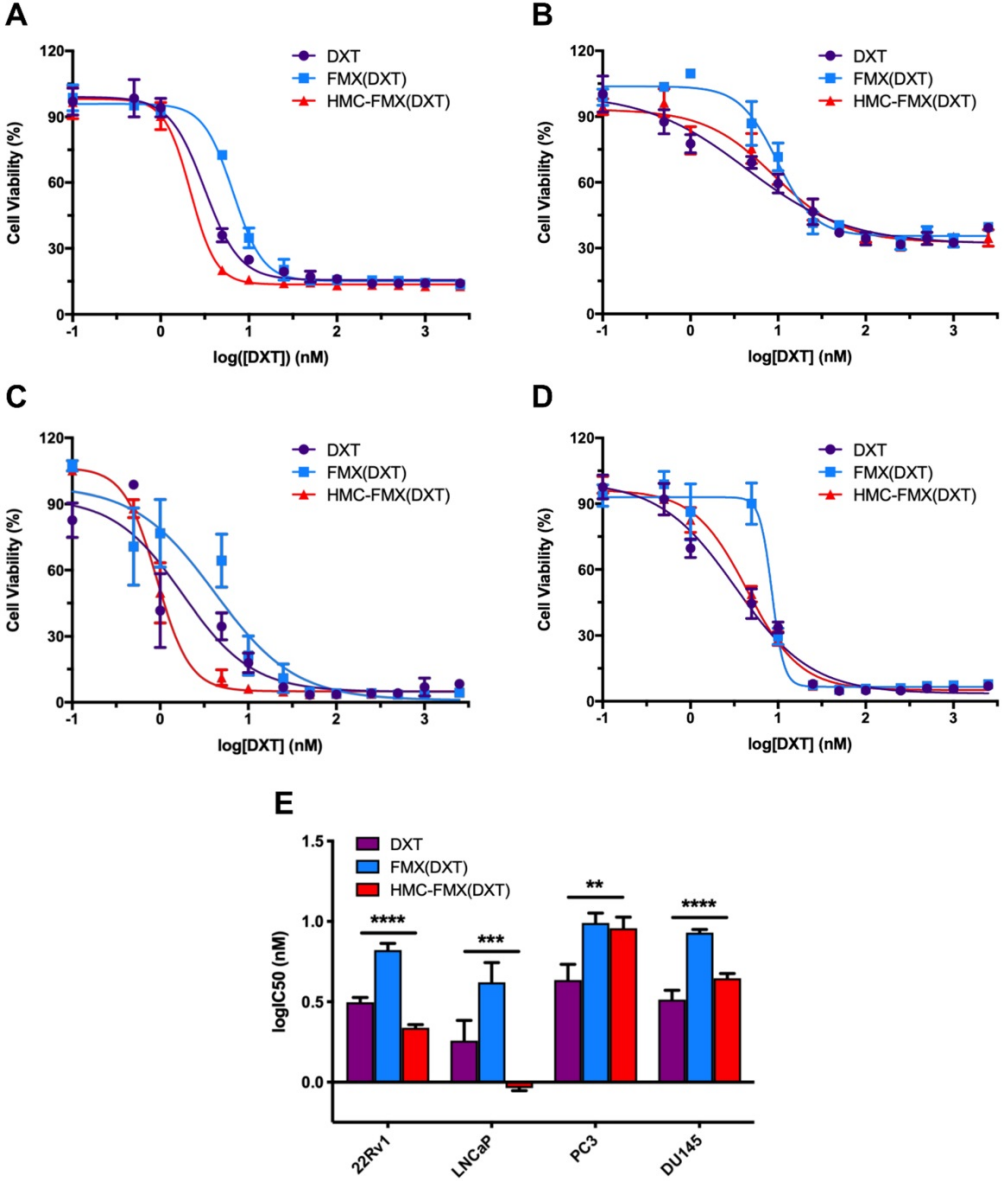
***In vitro* therapeutic efficacy of HMC-FMX(DXT) in PCa cells.** Representative IC_50_ curves for 22Rv1 (**A**), PC3 (**B**), LNCaP (**C**), and DU145 (**D**) cells treated with HMC-FMX(DXT), FMX(DXT), and DXT for 72 h. IC_50_ values of DXT-treated PCa cell lines are in the low nM range (**E**). **p < 0.01, ***p < 0.001, ****p < 0.0001, One-way ANOVA.

We next explored the *in vitro* cytotoxicity of HMC-FMX(DXT) toward 22Rv1, LNCaP, PC3, and DU145 PCa cell lines. Cells were treated with varying concentrations of DXT as either free drug, FMX(DXT) or HMC-FMX(DXT) (**Figures 5A-D**). Each cell viability-drug concentration curves were fit to a non-linear regression and the logarithm of the IC_50_ values for each cell line-treatment was determined (**Figure 5E**). Results showed that FMX(DXT) had a small but significantly higher IC_50_ value than DXT alone in all the cell lines studied (**Figure 5E and Table S3**). In contrast, HMC-FMX(DXT) had a reduced IC_50_ compared to FMX(DXT) and DXT alone in 22Rv1 and LNCaP, while HMC-FMX(DXT) values in in PC3 and DU145 were slightly higher when compared with FMX(DXT) or DXT alone. Regardless of these differences all the calculated IC_50_ values for HMC-FMX(DXT) were in the low nM range (<10 nM). Taken together, these results suggest that nanoparticle delivery of DXT does not mitigate its cytotoxicity to PCa cells.

**Figure 6:**
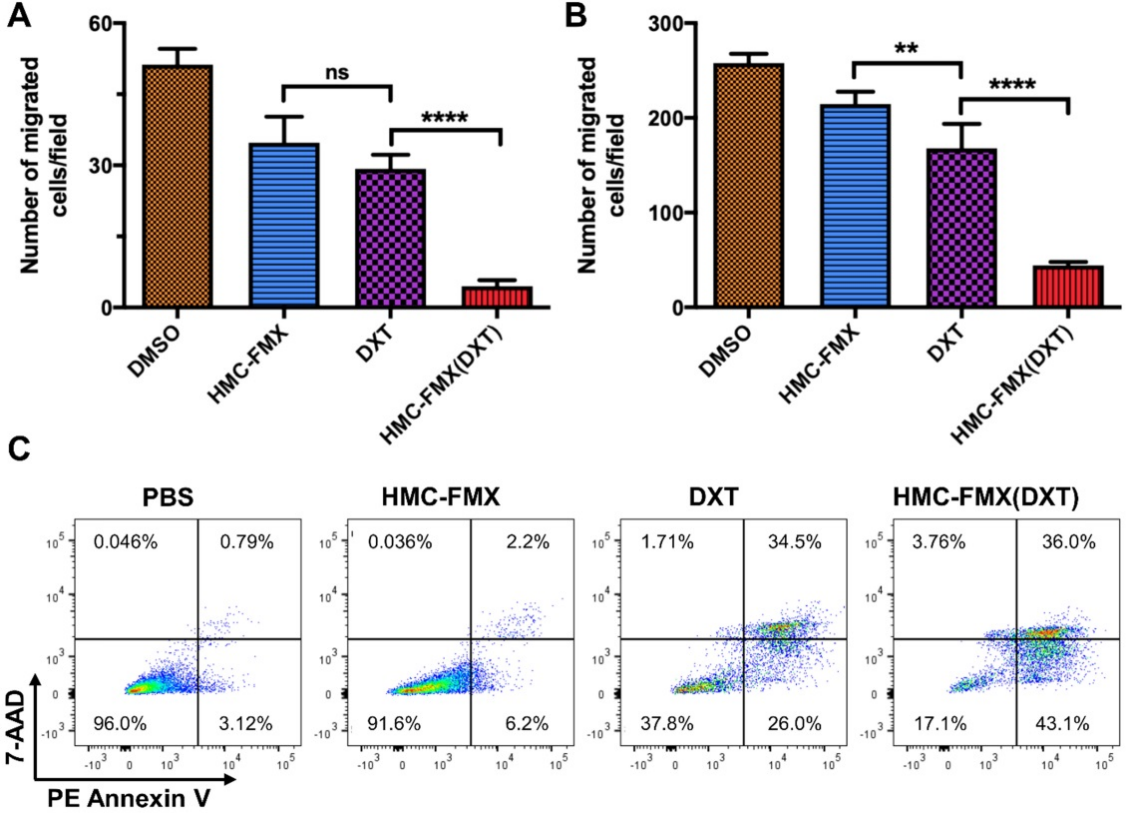
** HMC-FMX(DXT) inhibits migration and induces apoptosis in PCacells.** Average number of migrated LNCaP (**A**) and PC3 (**B**) cells in a transwell migration assay after 24 h of incubation. **p < 0.01, ****p < 0.0001, One-way ANOVA. Flow cytometry analysis (n=1) of 22Rv1 cells treated with PBS, HMC-FMX, DXT, and HMC-FMX(DXT) for 72 h (**C**).

We further validated HMC-FMX(DXT) efficacy in reducing migration of PCa cells. HMC-FMX(DXT) significantly inhibited the migration of LNCaP and PC3 cells, in contrast to both DXT- and HMC-FMX-treated LNCaP and PC3 cells (**Figures 6A and 6B**) in a transwell assay. The fact that HMC-FMX(DXT) has a significantly greater effect in reducing cell migration in both LNCaP and PC3 cultures suggest that HMC-FMX(DXT) could reduce metastasis *in vivo*. To test whether HMC-FMX(DXT) causes cellular apoptosis, we treated 22Rv1 cells with HMC-FMX(DXT) for 72 h, followed by flow cytometry analysis with 7-AAD/PE annexin V staining (**Figure 6C**). Results showed that in the PBS control and HMC-FMX treated cells, 96% and 91.6% of the cells were viable, with minimal amount of early apoptotic, late apoptotic or necrotic cells. The number of live cells were dramatically reduced in the DXT- and HMC-FMX(DXT)-treated cells to 37.8% and 17.1%, respectively, with a corresponding increase in the number of cells undergoing early (26% and 43.1%) late apoptosis (34.5% and 36%), or necrosis (1.71% and 3.76%). Taken together, these results indicate that HMC-FMX can deliver an anticancer drug such as DXT to PCa cells, reducing cell viability, causing apoptosis and inhibiting cell migration.

### HMC-FMX delivers anticancer drugs and reduces tumor volume in PCA mouse model

**Figure 7:**
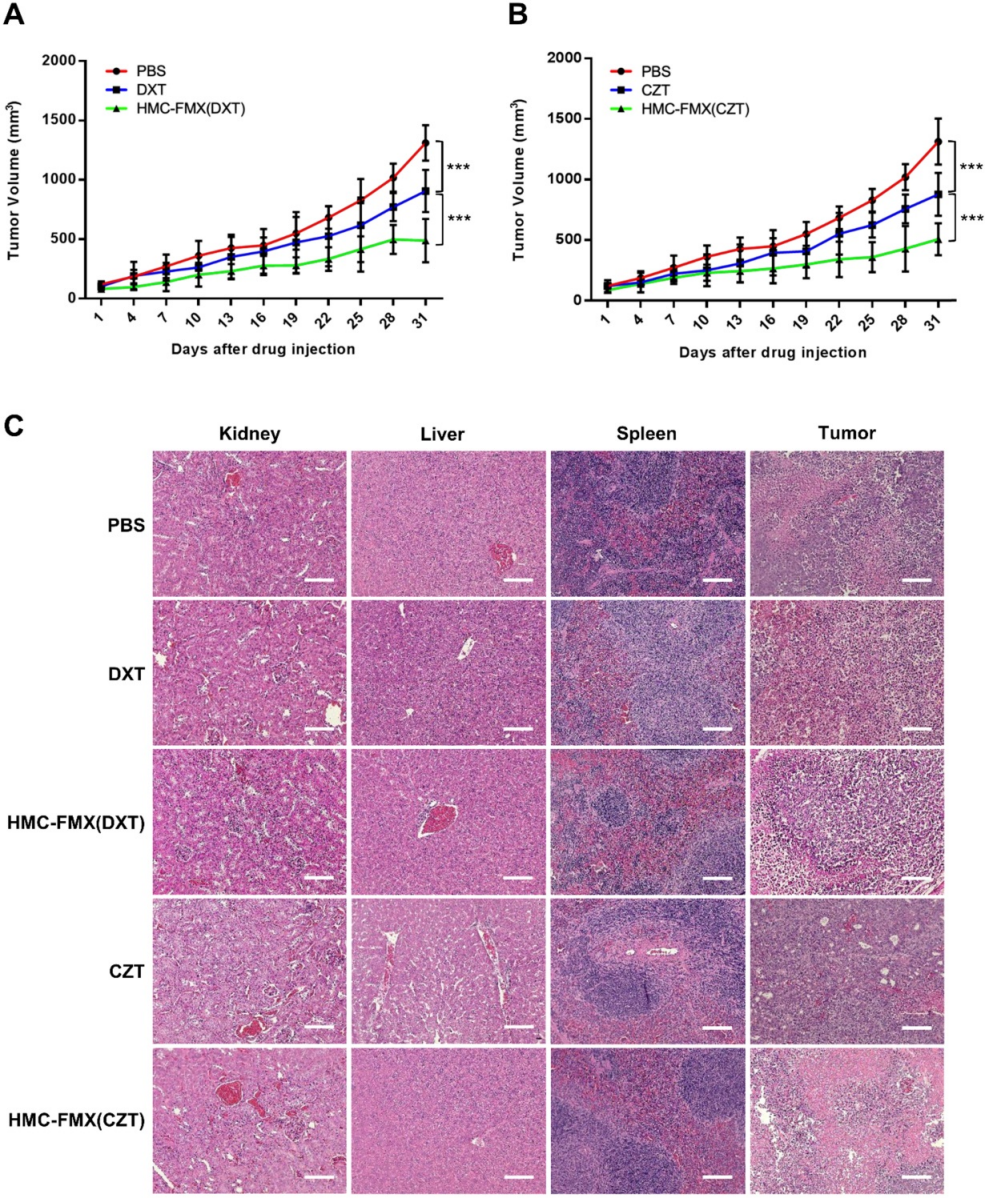
** Drug-loaded HMC-FMX reduces PCa tumor growth in mice.** Average tumor growth curves of mice bearing subcutaneous 22Rv1 prostate tumors with different treatment groups: PBS, DXT, and HMC-FMX(DXT) (**A**); PBS, CZT, and HMC-FMX(CZT) (**B**). ***p < 0.001, One-way ANOVA. Histopathological examination of kidney, liver, spleen, and tumor with and without nanoprobe treatment (**C**). Scale bars are 100 µm.

The ability of HMC-FMX(DXT) to reduce the growth of PCa tumors *in vivo* was tested in nude mice with subcutaneous 22Rv1 PCa tumors. In these experiments, we intravenously injected HMC-FMX(DXT) via tail vein to these mice. The prostate tumor volume was measured every three days until the end of the experiment. HMC-FMX(DXT) was able to slow down tumor volume over a period of 31 days, in contrast to DXT and PBS, where the tumor volume significantly increased (**Figure 7A**). Similarly, we tested the therapeutic efficacy of HMC-FMX encapsulating cabozantinib (CZT) in reducing the prostate tumor volume. CZT is an FDA-approved tyrosine kinase inhibitor for the treatment of medullary thyroid and advanced kidney cancers, that has also been evaluated for a range of other solid tumors including PCa tumors 42-47**.** In animal studies, CZT has been found to primarily affect cells of the tumor microenvironment, such as tumor associated macrophages and fibroblasts, while having a lesser effect in the actual PCa cells. The anti-proliferative effect of CZT was found to be two orders of magnitude greater on tumor-associated macrophages and fibroblasts compared to PCa cells, suggesting that CZT would affect cells of the tumor microenvironment more than the actual PCa epithelial cells 48. Extensive animal and human data support the use of CZT as an effective drug that affects PCa primary tumor as well as bone metastases 49-51. However, the high toxicity of the free drug, which limits its maximum tolerated dose and results in side effects, limits its clinical use. Thus, we examine whether CZT encapsulated to HMC-FMX can effectively reduce the growth of prostate tumors. We were able to successfully encapsulate CZT, resulting in an HMC-FMX(CZT) preparation with 67% encapsulation efficiency, similar to the encapsulation obtained in HMC-FMX(DXT) (**Table S2**). *In vivo* studies showed that HMC-FMX(CZT) had a similar effect to HMC-FMX(DXT) in reducing the growth of PCa tumors in mice (**Figure 7B**). Both HMC-FMX(DXT) and HMC-FMX(CZT) showed no toxicity to the kidney, liver, and spleen of mice implanted with subcutaneous 22Rv1 prostate tumors, when visualized by H&E staining (**Figure 7C**). Overall, these results suggest that both CZT- and DXT-loaded HMC-FMX are effective in reducing the prostate tumor volume in mice, and do not cause overall toxicity and damage to normal organs as seen upon administration of the free drugs.

## Discussion

In the current study, we report the development of a fluorescent nanoparticle-based image-guided system for both the intra-operative assessment of PCa tumor margins during surgery as well as postsurgical drug delivery system to treat recurrent disease. An accurate visualization of tumor margins during surgery is required to achieve complete removal of the PCa tissue. In addition, effective postsurgical chemotherapy treatment is equally needed to avoid recurrent and metastatic disease 32. Fluorescence imaging is the most promising approach for the intraoperative fluorescent image-guided visualization of PCa tumor margins and sentinel lymph node metastasis 2-4, 8, 51-53.

Our image-guided nanotheranostic platform is based on ferumoxytol (FMX) because of its current clinical use as an iron replacement medication, well-known pharmacokinetic properties, and ability to be easily modified with targeting ligands 20, 23, 24, 55. Ferumoxytol is emerging as a promising alternative to gadolinium-based MR contrast agents, because of its safety profile, biocompatibility, and FDA clearance. Most recently, it was found that Ferumoxytol, in contrast to common gadolinium-based agents, did not deposit at detectable levels in porcine brains at doses of 5-10 mg Fe/kg 56. Furthermore, FMX has been proposed as a nanoagent to screen those patients more suitable and responsive to a nanoparticle-based therapy, proposing its potential use in a personalized medicine setting 57, 58**.** In this scenario, patients could be first screened with HMC-FMX to identify those who could be more responsive to a HMC-FMX(Drug) therapy, by accessing tumor localization using MR imaging. Of a wide selection of ligands that one can choose to conjugate to FMX and facilitate targeting to PCa, we selected the heptamethine carbocyanine (HMC) ligand to conjugate to FMX (**Scheme 1**). HMC behaves as a dual cancer-targeting nanoagent and an NIRF nanoprobe, with excitation in 750 nm and emission at 800. The dual NIRF imaging and cancer-targeting ability of HMC is unique and upon conjugation yields an HMC-FMX nanoparticle with dual NIRF capabilities, as well as PCa targeting ability via OATPs.

The resulting HMC-FMX retained its magnetic and fluorescent properties (**Figure 1**), resulting in a monodisperse and stable nanoparticle suspension. HMC-FMX was able to internalize and fluorescently labeled various PCa cell lines *in vitro* (**Figure 2**). Such internalization was abrogated, when the cells were preincubated with OATP inhibitors suggesting that internalization was mediated by OATP. Meanwhile, upon administration of the HMC-FMX nanoparticle *in vivo*, specific localization to subcutaneous PCa tumors with corresponding fluorescent labeling was achieved using both a 22Rv1 or PC3 cancer model (**Figure 3**). In addition, HMC-FMX was able to localize to an orthotopic PCa tumor (**Figures 4B and 4C**), making the tumor fluorescently visible using either an IVIS or SIRIS camera. Minimal fluorescence was not observed throughout the animal or in any of the organs isolated, indicating selective tumor localization. Even though, accumulation of HMC-FMX in other organs still occurs (**Figure S4**), it does occur at levels low enough not to interfere with the identification of tumor borders by fluorescence imaging as seen in Figure 4. The orthotopic PCa tumor was detected intraoperatively by NIRF using SIRIS with clear delineation of the tumor margins (**Figure 4D**), which correlated with histological analysis. These results agree with previous reports that indicate HMC specifically targets PCa tumors, with minimal accumulation in other normal non-cancerous tissue 11, 16. Although other tissues are known to express various OATPs transporters, they express these transporters at a lower levels than in tumor tissue 10. However, the ability of FMX to accumulate in tumor via the enhanced permeability and retention (EPR) must also be considered as a possible mechanism that along with OATP active targeting, contributes to the retention of HMC-FMX in PCa tumors.

A unique feature of HMC-FMX is that it can encapsulate drugs within the carboxymethyl dextran coating of FMX allowing for sustained drug delivery upon tumor localization, as the rate of drug release is faster in tumor acidic pH (**Figure S5**). The encapsulation of DXT into HMC-FMX did not dramatically affect its cytotoxicity to PCa cell lines, with IC_50_ in the low nM range, comparable to the free drug. We also found that HMC-FMX(DXT) reduce the migration of both PC3 or LNCaP cells significantly more than the free drug (**Figure 6**). These results are significant as they suggest the potential use of HMC-FMX(DXT) to prevent or treat metastatic PCa. *In vivo* studies show a significantly slower growth of subcutaneous PCa tumor growth in mice injected with HMC-FMX(DXT), when compared with DXT alone or PBS (**Figure 7A**). Similar results were obtained with HMC-FMX(CZT) (**Figure 7B**). Our findings confirmed previous reports that FMX can deliver drugs to subcutaneous tumors in mice and furthermore that HMC facilitates the assessment of drug delivery to these tumors, while also aiding the visualization of tumor margins during surgery.

In summary, we have developed a multimodal and theranostics HMC-FMX nanoparticle that can be used to visualize PCa tumor margins using NIRF imaging during surgery and deliver chemotherapeutic drugs to these tumors post-surgery. The specific targeting of HMC-FMX is driven by the expression of OATPs in PCa cells. This simultaneous image-guided approach could potentially increase PCa treatment efficacy by facilitating maximum resection of PCa tissue within the prostate and enhancing chemotherapeutic drug accumulation in prostate tumors for an improved post-surgical treatment in patients with PCa.

## Supplementary Material

Supplementary figures and tables.

## Figures and Tables

**Table 1:** Physiochemical properties of HMC-FMX and FMX nanoprobes

Sample	Diameter*^a^* (nm)	Zeta-potential*^a^* (mV)	PDI*^a^*	Number of HMC per FMX*^b^*
FMX	23.2 ± 0.4	-8.2±0.7	0.10±0.01	--
HMX-FMX	37.0 ± 3.0	-11.8±0.3	0.32±0.03	40

a: Determined by dynamic light scattering (DLS); b: Determined by a standard curve based on UV-Vis spectroscopy of free HMC dyes in PBS.
