# Supplementary material for: Intraoperative assessment and postsurgical treatment of prostate cancer tumors using tumor-targeted nanoprobes
Source: Nanotheranostics. 2021 Jan 1;5(1):57–72. doi: 10.7150/ntno.50095 (PMC7738944; doi:10.7150/ntno.50095)
Supplement: Supplementary file 1 — Supplementary figures and tables. [file ntnov05p0057s1.pdf]

SUPPORTING INFORMATION

Intraoperative assessment and postsurgical treatment of prostate cancer tumors using tumor-targeted nanoprobes

James Teh[1], Manisha Tripathi[1,6], Derek Reichel[1], Bien Sagong[1], Ricardo Montoya[1], Yi Zhang[2], Shawn Wagner[2], Rola Saouaf[4,5], Leland W. K. Chung[3], J. Manuel Perez[1,4,5]

[1]Department of Neurosurgery, Cedars-Sinai Medical Center, Los Angeles, CA 90048
[2]Biomedical Imaging Research Institute, Cedars-Sinai Medical Center, Los Angeles, CA 90048
[3]Department of Medicine, Uro-Oncology Research Program, Cedars-Sinai Medical Center, Los Angeles, CA 90048
[4]S. Mark Taper Foundation Imaging Center, Cedars-Sinai Medical Center, Los Angeles, CA 90048
[5]Samuel Oschin Comprehensive Cancer Institute, Cedars-Sinai Medical Center, Los Angeles, CA 90048
[6]Current address: Department of Cell Biology and Biochemistry, Texas Tech University Health Sciences Center, Lubbock, TX 79430

Corresponding Author
*Email:jmanuel.perez@cshs.org

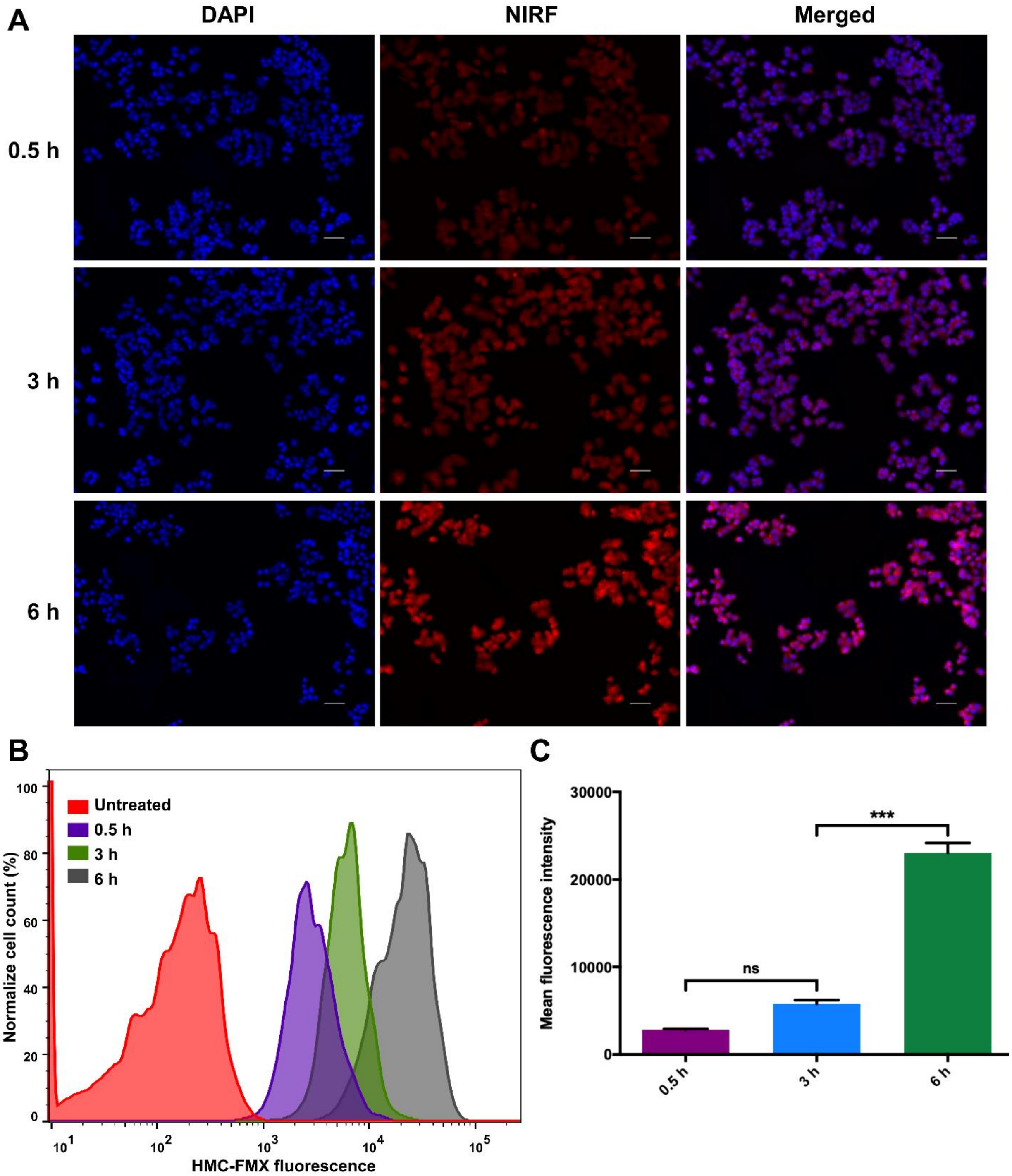

Figure S1. Time-dependent fluorescence intensity of HMC-FMX following cellular internalization. Representative fluorescence images of 22Rv1 cells at different incubation periods (30 min, 3 h, and 6 h) treated with HMC-FMX [**A**]. HMC-FMX fluorescence in 22Rv1 cells analyzed by flow cytometry at 30 min, 3 h, and 6 h [**B**]. Average fluorescence intensity measurements of 22Rv1 cells treated with HMC-FMX at different time periods [**C**]. In these images, blue represents nuclei, and red represents HMC-FMX. Scale bars are 50 μm. Magnification: 20x. ***$p < 0.001$, One-way ANOVA.

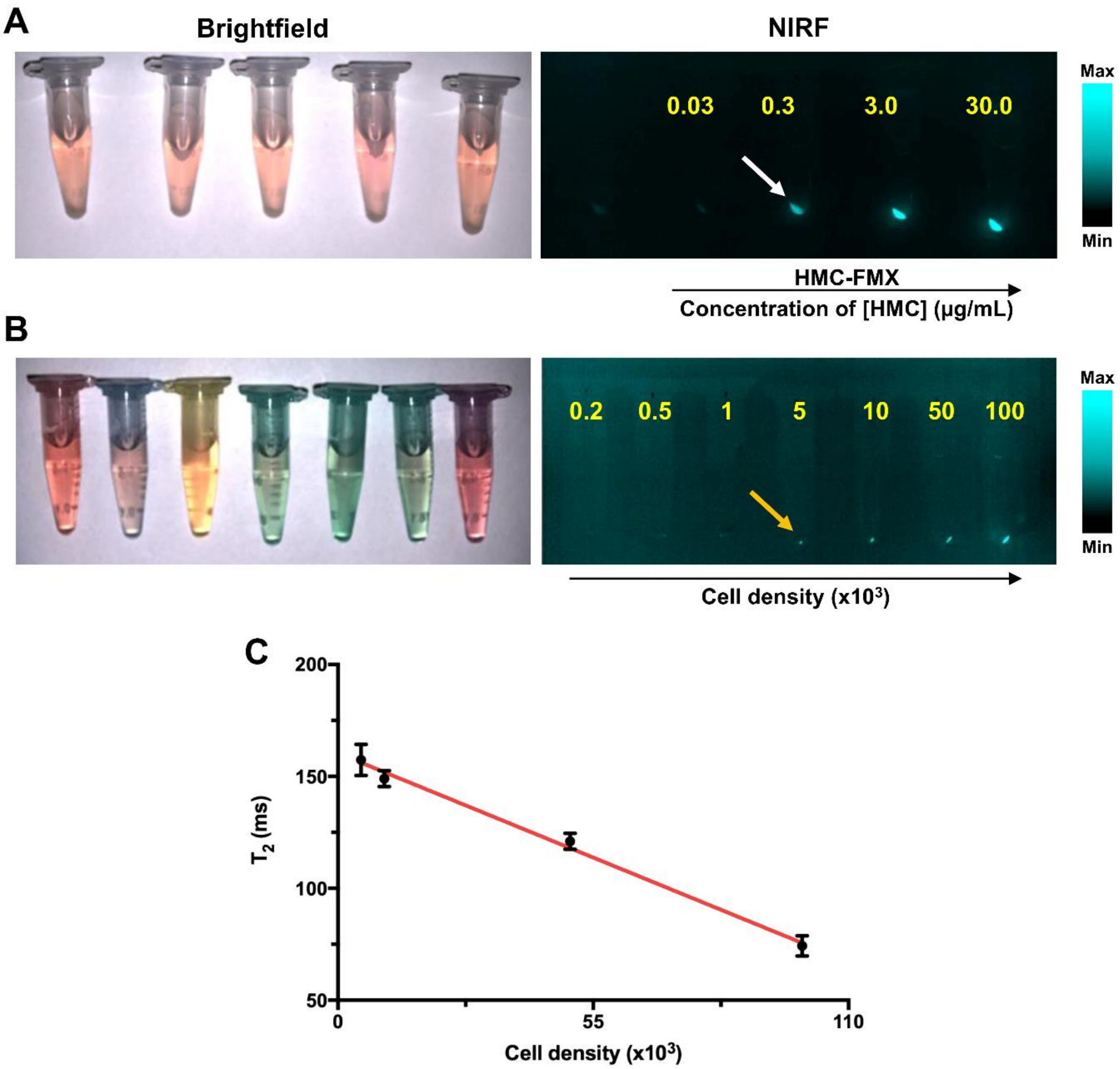

Figure S2. Near infrared fluorescence (NIRF) and magnetic resonance (MR) characterizations of HMC-FMX in prostate cancer cells. Brightfield and NIRF images show pellets of LNCaP cells, after treated with vary concentrations of HMC-FMX for 24 h [**A**]. White arrow indicates minimum HMC-FMX fluorescence signal detected at 0.3 µg/mL [HMC]. Brightfield and NIRF images show pellets of different LNCaP cell densities, after treated with HMC-FMX (0.3 µg/mL [HMC]) for 24 h [**B**]. Orange arrow indicates minimum HMC-FMX fluorescence signal detected in 5 X 10^3 LNCaP cells. T_2 relaxation time of HMC-FMX incubates with different LNCaP cell densities [**C**].

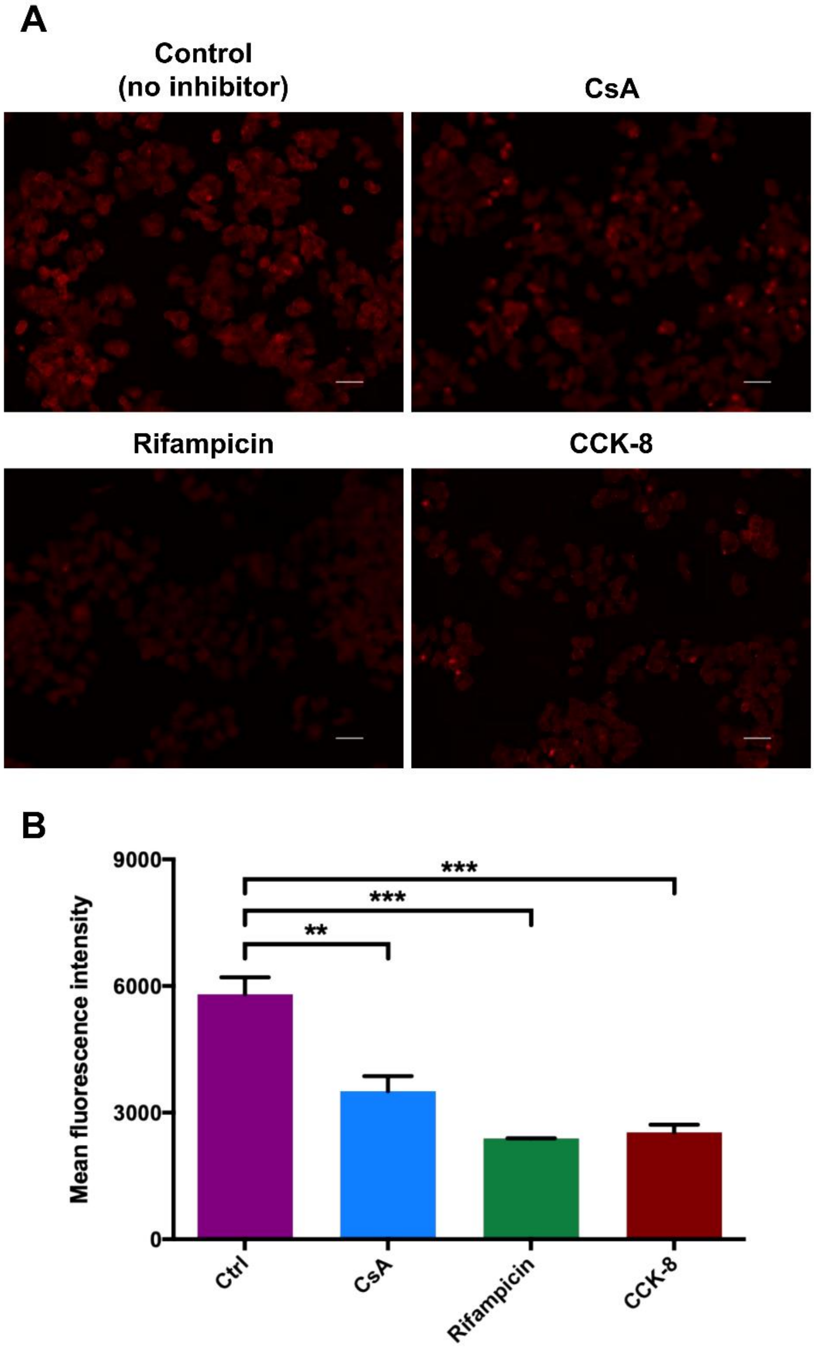

Figure S3. OATP inhibitors affect cellular uptake of HMC-FMX. 22Rv1 cells are pretreated for 3 h with OATP inhibitors – CsA (20 μM), rifampicin (25 μM), and CCK-8 (20 μM) – and then treated with HMC-FMX for 3 h. Fluorescence microscope images [**A**] and average fluorescence intensity measurements [**B**] show decreased HMC-FMX fluorescence in 22Rv1 cells after pretreatment with OATP inhibitors. Mean fluorescence intensity in 22Rv1 cells measure by flow cytometry. Scale bars are 50 μm. Magnification: 20x. $^{**}p < 0.01$, and $^{***}p < 0.001$, One-way ANOVA.

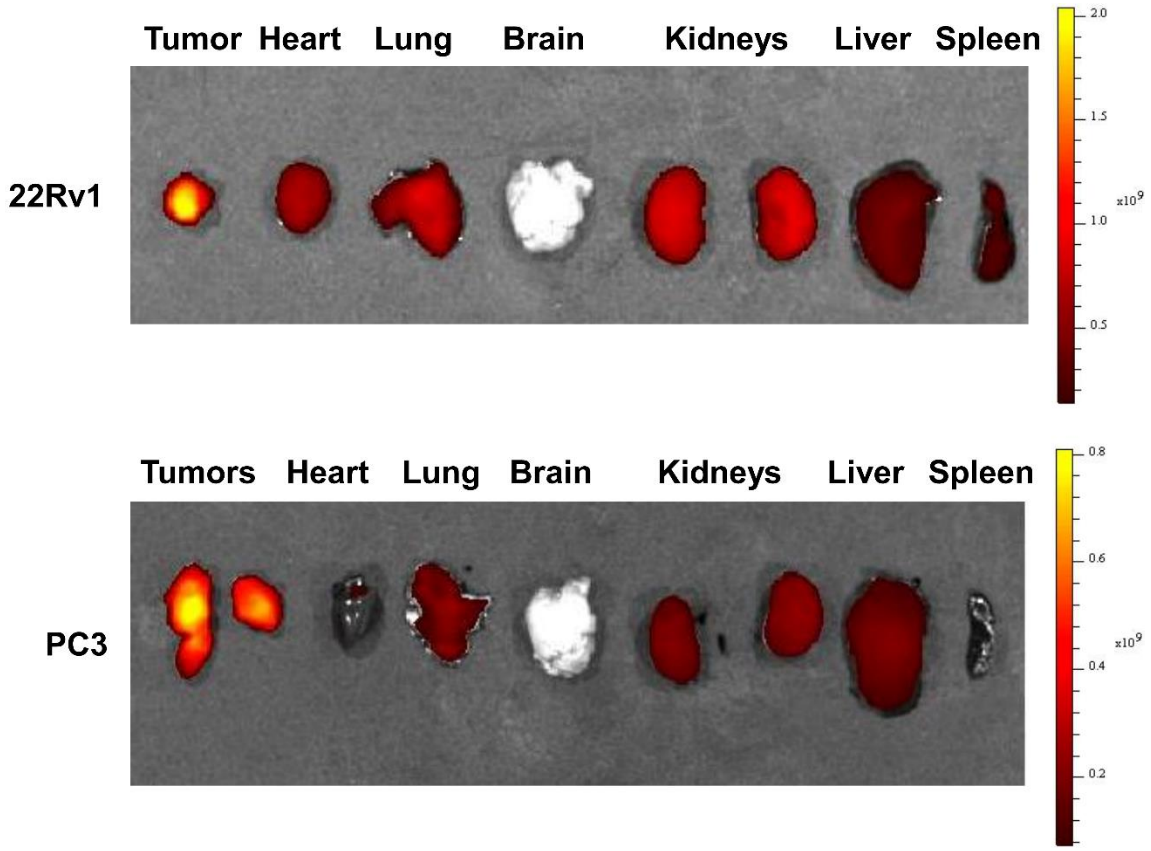

Figure S4. ***Ex vivo*** **NIRF imaging of mouse organs and tumors.** Fluorescence distribution in the tumors, heart, lung, brain, kidneys, liver and spleen, after 72 h post-injection with HMC-FMX.

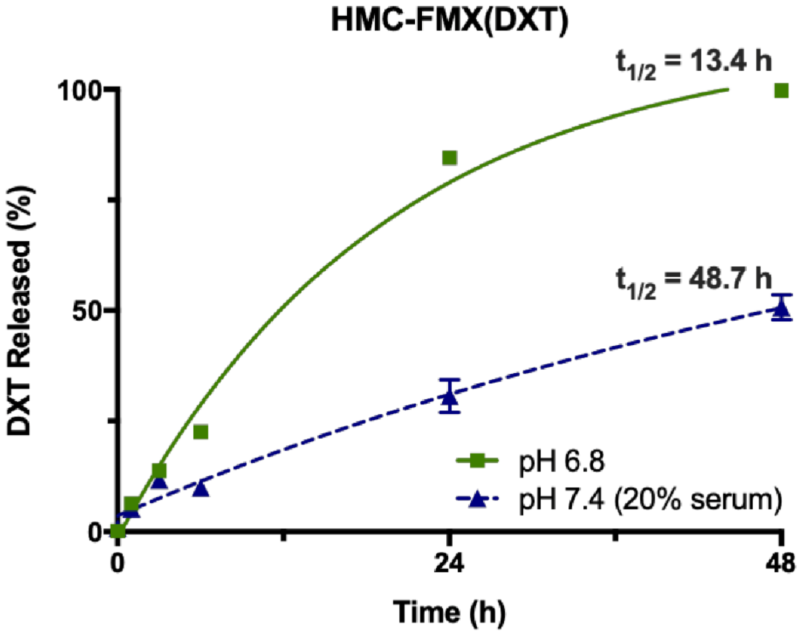

Figure S5. Rate of drug release from HMC-FMX. Release profile of HMC-FMX(DXT) in PBS quantified at pH 6.8, and at pH 7.4 with 20% serum. The DXT release half-life in pH 6.8, and pH 7.4 with 20% serum are 13.4 h and 48.7 h, respectively.

Table S1. Relaxivity measurements of FMX, HMC-FMX, and HMC-FMX(DXT) nanoprobes. Differences in r_1, r_2 and r_2/r_1 were not statistically significant (p > 0.05).

Sample	r_1 (L/mmol*s)	r_2 (L/mmol*s)	r_2/r_1
FMX	9.1 ± 0.1	82.3 ± 1.5	9.0
HMX-FMX	8.5 ± 0.7	85.2 ± 1.6	10.0
HMC-FMX(DXT)	8.3 ± 0.6	81.2 ± 1.3	9.8

Table S2. Characterization of drug-loaded HMC-FMX nanoprobes

Sample	Diameter[a] (nm)	Zeta-potential[a] (mV)	PDI[a]	[Drug][b] (mM)	% [Drug] Encapsulation	% [Drug] Loading
HMX-FMX	37.0 ± 3.0	-11.8 ± 0.3	0.32 ± 0.03	--	--	--
HMX-FMX(DXT)	40.0 ± 4.5	-11.2 ± 0.9	0.33 ± 0.08	0.615	62	30
HMC-FMX(CZT)	41.3 ± 2.7	-11.7 ± 0.4	0.37 ± 0.12	0.637	67	20

[a]Determined by dynamic light scattering (DLS). [b]Determined by a standard curve based on HPLC quantification of free drugs.

Table S3. IC50 values of prostate cancer cells treated with DXT, FMX(DXT), and HMC-FMX(DXT). Results are reported as mean with 95% confidence intervals in brackets below.

	Cell lines			
Treatment	22Rv1	LNCaP	PC3	DU145
DXT (nM)	3.14<br>[2.71 - 3.64]	1.81<br>[1.01 - 3.27]	4.32<br>[2.60 - 7.18]	3.26<br>[2.40 - 4.31]
FMX(DXT) (nM)	6.63<br>[6.08 - 7.24]	4.18<br>[2.36 - 7.38]	9.78<br>[8.49 - 11.3]	8.51<br>[7.83 - 8.71]
HMC-FMX(DXT) (nM)	2.18<br>[1.93 - 2.47]	0.92<br>[0.85 – 0.98]	9.07<br>[6.86 - 12.0]	4.43<br>[3.76 - 5.12]
